# Investigation on the Cancer Invasion and Metastasis of Skin Squamous Cell Carcinoma by Raman Spectroscopy

**DOI:** 10.3390/molecules24112059

**Published:** 2019-05-30

**Authors:** Xu Zhang, Fan Yu, Jie Li, Dongliang Song, Heping Li, Kaige Wang, Qingli He, Shuang Wang

**Affiliations:** 1 Institute of Photonics and Photon-Technology, Northwest University, Xi’an 710069, China; 13709146215@163.com (X.Z.); 15771915828@163.com (F.Y.); LJ15891397079@163.com (J.L.); SONGNWU@163.com (D.S.); 18291861688@163.com (H.L.); kaigewang@126.com (K.W.); 2 Department of Physics, Northwest University, Xi’an 710069, China; heqingli@nwu.edu.cn

**Keywords:** squamous cell carcinoma, Raman microspectroscopy, skin carcinogenesis, cancer metastasis

## Abstract

Raman spectroscopy facilitates accurate and minimally invasive investigation on biomedical samples to reveal their molecular-level biological information. In this work, the cancer field effects of squamous cell carcinoma (SCC) tissues were illustrated by Raman microspectroscopy. Referenced with hematoxylin and eosin (H&E) stained microscopic images, the biochemical variations during SCC progress were meticulously described by the Raman spectral features in different pathological areas of two lesion types, including the biochemical changes in collagen, lipids, DNA, and other components of SCC diffusion and metastasis. The experimental results demonstrated that the intensities of the Raman peaks representing collagen (853, 936, and 1248 cm^−1^) were decreased, whereas the intensities of peaks corresponding to DNA (720, 1327 cm^−1^) and lipids (1305 cm^−1^) were increased significantly in cancerous lesions, which testified that SCC originates from the epidermis and invades the dermis gradually. The achieved results not only described the molecular mechanism of skin carcinogenesis, but also provided vital reference data for in vivo skin cancer diagnosis using Raman spectroscopy.

## 1. Introduction

Skin cancer is one of the common malignant tumors, mostly due to excessive exposure to UV radiation, chemical carcinogens, and environmental contamination [1,2,3,4]. The incidence of skin cancer has been increasing in many countries with Caucasian, Mongoloid, and Negroid populations [5,6]. Approximately 20% of American white people are estimated to have at least one skin cancer occurrence during their lifetime, in relation to their geographical location and lifestyle [7,8]. According to the statistics, squamous cell carcinoma (SCC) has the highest incidence in China, accounting for 90% of skin cancer cases [9,10]. The morbidity of basal cell carcinoma (BCC) is only one-tenth severe compared to squamous cell carcinoma [10]. Malignant melanoma is the rarest but most severe among the three types of skin disease, and spreads immediately after onset. Pathologic examination and surgical procedures are still the gold standards of diagnosis and treatment in skin cancer, but their accuracies are highly dependent on the experiences of the operators. Therefore, it is necessary to explore a fast, accurate and minimally invasive analysis in early skin cancer detection and pathophysiological investigations. 

The Raman microspectroscopy used in this study detects sub-cellular components using the spectral fingerprints of molecules based on their characteristic vibrations [11,12,13,14]. Previous studies have proved that Raman spectroscopy is very sensitive to the basic biochemical variations of cancerous cells in the digestive system, breast cancer, and the female reproductive system in histomorphology at the microscopic level, providing a solid experimental basis for the early diagnosis of cancer [15]. In our previous work, we carried out a spectral histopathology study to describe the correlation between the biochemical profile and histological architecture of ex vivo healthy human skin tissue [16]. Since all disease states are caused by fundamental changes in cellular and/or tissue biochemistry, variation in skin composition during its carcinogenesis can be studied qualitatively and quantitatively. Hence, this study was aimed to understand the biopathological features of SCC from the perspective of Raman spectroscopy, particularly for invasion activity during its pathological course. After different lesion regions were located in unstained sections, referenced with hematoxylin and eosin (H&E) staining images, the spectral features of different tissue structures were realized and identified as carcinogenesis for relevant indicators for in vivo diagnosis and prognosis of skin cancer.

## 2. Results

**Figure 1:**
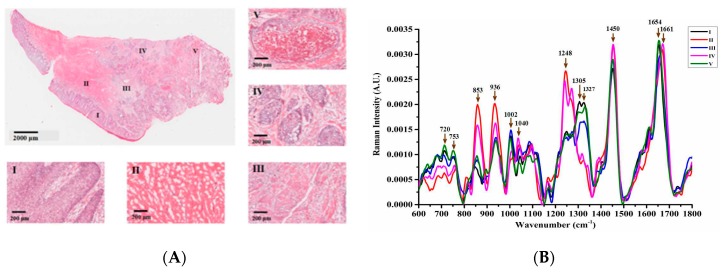
(**A**) shows the hematoxylin and eosin (H&E) stained image of the primary squamous cell carcinoma (SCC) lesion. In that, the symbols I–V mark the different lesion area. The magnified image of the typical epidermis is marked as region I; collagen fiber reticulation in region II indicates a typical dermal layer structure; region III lies between the dermis and the mature tumor mass; the neoplasia occurring in the dermis area IV; and necrotic tissue mass is presented in region V; (**B**) shows normalized mean spectra of different areas for primary SCC lesion, in which the arrows point to some prominent Raman peaks.

SCC originates from the keratinocytes of the epidermis and its appendages (hair follicle funnel, sebaceous duct, and terminal sweat tube) with a histologically distinct form [17]. As shown in Figure 1A, a typical low-grade skin SCC lesion was pathologically verified with irregular lumps, which had already grown downwards through the basement membrane and invaded the dermis. The whole tissue section was then divided into five different morphological regions named I, II, III, IV, and V, whose partial micro-enlargement images are displayed in Figure 1A, respectively. Region I was mainly located in the stratum germinativum layer of the epidermis with histological features of columnar and stratum spinosum cell distribution. The reticular dermis in region II was featured with reticulation architectures and densely packed collagen fibers. Region III lay in the center of the sampled section, which is a contact area between the dermis and mature tumor mass with some cancer cells distributed. In region IV, the tumor mass could be clearly separated from the proliferating and metastasizing cancer. Region V exhibited histological characteristics of a necrotic tissue mass, where dead cells were located. According to the histopathological analysis, the invasion direction of the cancer cells was concluded to be roughly from the right to the left, presenting the severity of neoplasia as gradually deepening.

After histological evaluation, the spectra from 30 randomly selected points were acquired in each separated region. The spectral feature for each region was presented with the mean value of acquired spectra from 600 cm^−1^ to 1800 cm^−1^ in Figure 1B, whose biochemical assignments are listed in Table 1. For a visual understanding, the acquired spectra could be summarized into two groups based on spectral variations from 1200 to 1400 cm^−1^. The higher spectral intensities at 853, 936, 1040, 1248, 1450 and 1661 cm^−1^ were presented in regions II (red line) and IV (pink line), whereas the mean spectra in region I (black line), III (blue line), and V (green line) exhibited a more conspicuous spectral feature at the 720, 753, 1002, 1305, 1327 and 1654 cm^−1^ bands. 

Except for the main lipid bands at 1450 cm^−1^ (CH2 scissoring of proteins and lipids), the other main constituent of the skin dermis was observed as double Raman bands around 853 and 936 cm^−1^, originating from the amino acid side chain vibrations of proline and hydroxyproline, as well as a υ(C-C) vibration of the collagen backbone. Besides that, a strong Raman band at 1248 cm^−1^ in both the II and IV regions were usually derived from the υ(CN) and δ(NH) amide III of collagen. So, the intensities of 853, 936 and 1248 cm^−1^ indicated that regions II and IV had a similar constitution of a healthy dermis skin layer. On the contrary, the epidermis, which consisted of 95% keratinocytes [31], exhibited the lowest collagen contribution, as shown by the spectrum of region I. The intensity variations of collagen Raman bands are attributed to the composition differences between the dermis and non-dermis tissues, and indicated dermis structure destruction during SCC pathological progress, such as a lowered collagen content in regions III and V. 

The Raman band at 720 cm^−1^ was attributable to the C-N vibration of nucleic acids, indicating a higher density of cells or nuclear distribution in regions I, III, and V than that in regions II and IV. The relative intensity of the 1327 cm^−1^ band derived from epidermal cells, which was due to the CH3-CH2 wagging mode in the purine bases and phospholipids of the DNA, showed a similar intensity variance with the 720 cm^−1^ Raman band. The peak at 753 cm^−1^ was associated with the symmetric breathing of tryptophan. As reported by Devpura et al. [17], there is a significant increase of tryptophan in SCC tissue compared with that in normal skin tissue, and tryptophan may be a contributing factor to further tumor progression from the epidermis to dermis. Tankiewicz et al. [32] also demonstrated changes in tryptophan metabolism reflected by the increased content of tryptophan and its metabolites in patients with oral SCC. The peak at 1002 cm^−1^ was caused by changes in the ring-breathing vibrational mode due to phenylalanine. It is thought that the content variation of phenylalanine is linked to inflammation and immune activation during cancer progression. The resulting oxidative stress could impair the activity of phenylalanine (4)-hydroxylase (PAH) and tryptophan oxygenase, which then results in increased phenylalanine and tryptophan concentrations [33]. 

The band at 1305 cm^−1^ is normally assigned to twisting and wagging vibrational modes between carbon and two hydrogen ions (CH_2_) in lipid molecules. This peak was almost absent in regions II and IV but was abundant in the cancerous epidermis (region I) as well as in the diseased regions of III and V, which further demonstrated the SCC pathology originating from the epidermis and invading the dermis. Whereas the 1661 cm^−1^ peak related to the amide I vibrational modes from collagen, the 1654 cm^−1^ peak was from thymidine, guanine, cytosine (ring-breathing modes of the DNA/RNA bases) and amide I with a lipid assignment, which signified the constitution difference between the cancerous group (region I, III and V) and the non-cancerous group (region II and IV). 

The Raman band around 1040 cm^−1^, which presented in regions II and IV was not typically identified. Zhiwei et al. [34] and Shim et al. [35] found that this spectral feature in ex vivo measurements might be attributed to formalin fixation; however, because we used fresh tissue samples in our study, formalin Raman contaminations could be excluded. An in vivo skin Raman study [32,33] has testified that this peak can result from albumin oxidation and investigated this attribution by measuring purified cysteic acid, which is a common amino acid in organisms causing delinking of albumin disulfide bonds. Although albumin could be used as a biomarker for oral SCC and chronic periodontitis [36], it still remains to be determined whether this Raman spectral feature is related to the pathological course of skin SCC or is an overlap with its nearby peaks around 1032 cm^−1^ as C-C stretching modes of keratin. 

**Figure 2:**
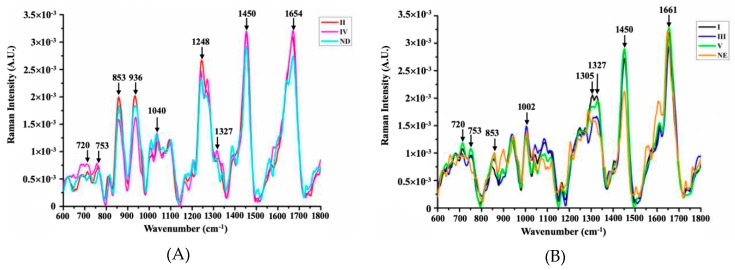
Comparison of Raman spectra from the II and IV regions with the normal dermis (**A**) and spectral comparison between regions I, III, IV and the normal epidermis (**B**). In that, ND is the abbreviation for normal dermis, and NE is for normal epidermis.

For a detailed analysis, spectra from healthy human epidermis and dermis were adopted to compare the obtained two groups of spectra, as shown in Figure 2. According to the analysis of Figure 1B and Figure 2A, the levels of collagen (853, 936, 1248 cm^−1^) in region IV were significantly lower than those in normal dermis, whereas the levels of nucleic acids (720, 1327 cm^−1^) and lipids (1450, 1654 cm^−1^) were slightly increased. This indicated slight changes in the substances in region IV, which may be due to the presence of cancer cells. As shown in Figure 2B, the spectral line patterns of I, III, and V are similar to those of the normal epidermis. The green line (V) had the largest intensities at 720, 753 and 1450 cm^−1^ compared with the regions I and III. It could thus be concluded that in the course of SCC neoplasia, collagen content declines, whereas the DNA and lipid content increases during the invasive action of SCC. 

Based on the spectral features in different lesion regions, the complete process of SCC tumor metastasis could be concluded. The skin epidermis (I) was firstly transformed, followed by invasion of the dermis; therefore, region III—carrying tumor cells—further advanced into the dermis to overcome region II, with the result being that region II was transformed to region IV and eventually to V. Both regions III and V were derived from the same tissue as the epidermis (I), which originated in the lower right outermost position. Region V was mostly a necrotic tumor mass with no metastasis, which could be considered as the highest level of tumor growth in this case. 

**Figure 3:**
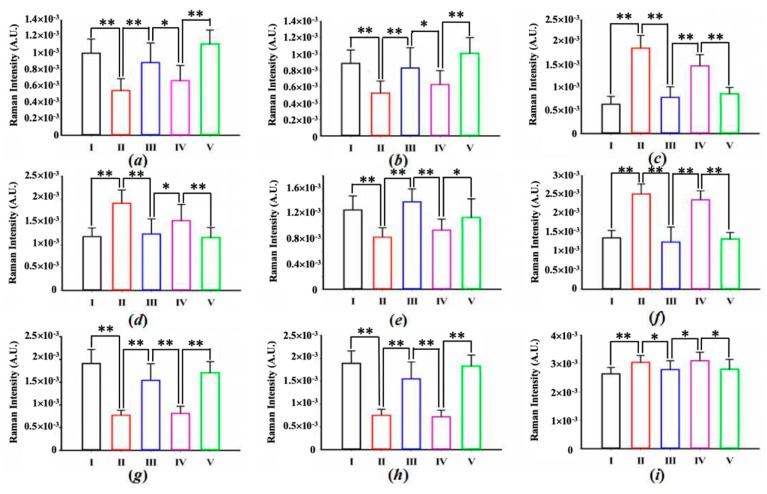
Intensity measures for each kind of composition. (**a**) 720 cm^−1^; (**b**) 753 cm^−1^; (**c**) 853 cm^–1^; (**d**) 936 cm^–1^; (**e**) 1002 cm^–1^; (**f**) 1248 cm^−1^; (**g**) 1305 cm^−1^; (**h**) 1327 cm^−1^; (**i**) 1450 cm^−1^. Take the spectra intensity from region I as the reference, one way ANOVA was followed by Tukey’s honest significant difference (HSD) post hoc multiple comparison tests. The values are presented as mean + standard error of the mean (*n* = 20 in each group). * *p* < 0.05, ** *p* < 0.01.

In order to determine the degree of variability among the characteristic Raman spectra of each type of measured area, a one way ANOVA was performed to detect nine statistically different peaks (significance level *p* < 0.05), as shown in Figure 3. Figure 3c,d,f, representing collagen bands 853, 936 and 1248 cm^−1^, respectively, all clearly show that the spectral contribution in regions II and IV was higher than that in the other three regions. The intensities of these peaks were all higher in region II than in region IV, indicating that region IV was undergoing a slight change, i.e., the collagen contents were decreasing and the dermis structure was vanishing at the same time. On the contrary, at positions 720 (a), 753 (b), 1002 (e), and 1305 cm^−1^ (g), the intensities of region IV were found to be slightly higher than those of region II, which proved that region IV experienced a more serious cancer invasion than that in region II. In regions I, III, and V, the higher intensities for peaks at 720 (a) and 1327 cm^−1^ (h), indicated a higher degree of cell or nuclear aggregation in the tumor. In addition, the intensity discrepancy of 1305 cm^−1^ between regions I, III, V and regions II, IV was also very significant (g), which further demonstrated lipid aggregation in the cancerous area. Among regions I, III, and V, the intensities of peaks at 720, 753, 1305, and 1327 cm^−1^ were the lowest in region III. Due to the special location of region III in the sample, it was affected by the dermal layer during its invasion into the dermis, resulting in several characteristic peaks decreasing temporarily, but the intensities of these peaks would increase after III conquers IV. 

**Figure 4:**
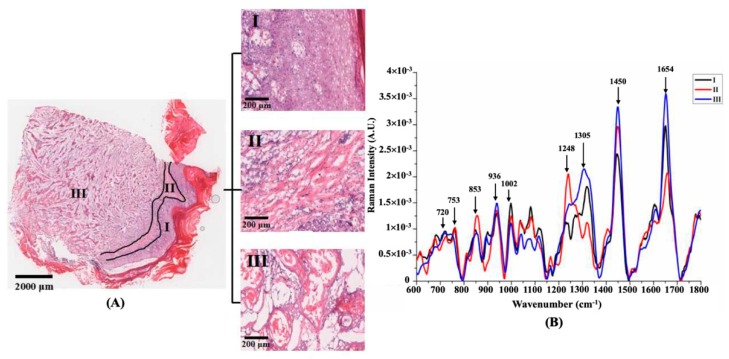
(**A**) The H&E stained specimen of the second SCC tissue, the symbols I, II, and III marks the epidermis, dermis, and cancerous area, whose partially magnified images are displayed. (**B**) The spectral comparison of the three regions.

Figure 4 shows an H&E micrograph of a grade 2 SCC tissue section, in which the cancerous tissue is distributed in a cord-like fashion. Because there were more intact epidermis and stratum corneum in the lower right with the lower left epidermis being severely absent, we speculated that the tumor metastasis of this sample developed from the bottom left to right. Figure 4A can be divided into three regions (region I, II and III) as presented individually next to the complete microscopy image. The cancerous region (III) showed a similar spectral shape to that of the epidermis (I), while the spectral characteristics of region II were still similar to those of healthy skin dermis, which provided good proof for the invasion direction of the cancer cells. Figure 4e shows that the peaks of 853 and 1248 cm^−1^ in the red line (II) were significantly higher than the other two areas, indicating abundant collagen distribution. The collagen content was greatly reduced in region III, which could be proved by the typical characteristic peak of collagen at 1248 cm^−1^. The broad peak from 1300 cm^−1^ and 1330 cm^−1^ showed a decreasing trend from region III to II, indicating the aggregation of lipids and DNA in the cancerous areas. The discrepancy in phenylalanine (1002 cm^−1^) intensity in each region appeared to present a different pattern compared to Figure 1B, showing a higher content in region II than in region III. The reason may be that the secondary SCC had a less severe inflammatory reaction compared to the primary SCC. The Raman peak at 1654 cm^−1^, representing amide I, was very strong in region III, but the same type of peak at 1661 cm^−1^ was unremarkable in region II, which further indicated that after the transformed epidermis invaded, the constitution and structure of the dermis would gradually diminish. the accumulation of large numbers of SCC tumor cells results in increased nucleoli and decreased cytoplasm, leading to an increased nucleo-cytoplasmic ratio [18]. The intensities of the Raman peaks representing DNA (720, 1327 cm^−1^) and tryptophan (753 cm^−1^) in regions I and III were slightly higher than those in region II, which exhibited a similar variation tendency with the primary SCC, as shown in Figure 2B. Whereas, a higher contribution of proteins (853 and 1248 cm^−1^) could be attributed to: (i) Uncontrolled and abnormal cell proliferation, (ii) cell division, and (iii) migration in the malignant tumor [37,38]. Since the identification of biomarkers with Raman spectroscopy could be useful to understand the physiology and biochemical progression in carcinogenesis, DNA, protein and lipid contents can play an important role as biomarkers, based on the reported spectral similarities in both two cases.

## 3. Materials and Methods

### 3.1. Sample Preparation

Human SCC skin tissues were bought from Alenabio company (Xi’an, China); they were collected from autopsies using IRB (Institution Review Board) and HIPAA (Health Insurance Portability and Accountability Act) approved protocols, and further approved for commercial product development. Based on a broader histological classification, a grade 1 SCC lesion was collected from the calf skin of a 51-year old female patient and a grade 2 SCC lesion was from the heel of a 50-year old male patient. Moreover, normal skin tissues were obtained from the foot of a 25-year old male. Immediately after tissue collection, unwashed samples were embedded in optimal cutting temperature (OCT) medium (Surgipath^®^ FSC 22^®^, Leica Biosystems Inc., Buffalo Grove, IL, USA) and frozen in liquid nitrogen to preserve their native morphology. Transverse sections of 20 μm thickness were prepared on gold-coated glass substrates (BioGold^®^ 63479-AS, Electron Microscopy Sciences Inc., Hatfield, PA, USA) for spectroscopic measurements [39,40]. Consecutive 5 µm thick sections were H&E stained for pathological analysis in consultation with a professional physician and then used as a reference to locate different lesion regions in the unstained sections. The frozen sections were kept in an acetone cooling bath for dehydration and stored at −80 °C until usage. The sections were allowed to thaw for 30 min prior to spectroscopic analysis. 

### 3.2. Raman Microspectroscopy System

Micro-Raman spectra were obtained with an Alpha 500R confocal Raman microspectroscopy system (WITec GmbH, Germany) coupled with a helium-neon (He-Ne) continuous 633 nm laser beam (35 mW @ 633 nm, Research Electro-optics, Inc., USA). The excitation laser beam was collimated into a 20× objective lens (NA = 0.85, N-Achroplan, Zeiss, Germany) for Raman excitation. Raman photons were collected by the same objective lens and transmitted through a holographic edge filter to a multi-mode optical fiber (50 μm diameter) to the spectrometer (UHTS300, WITec GmbH, Germany), which was equipped with a resolution about 3 cm^−1^ over a spectrum range of 0–2400 cm^−1^. The spectra were recorded using a back-illuminated, deep depletion CCD camera containing 600 × 200 pixels (Du401A-BR-DD-352, Andor Technology, UK) working at −60 °C. The spectral data were acquired point-by-point over each kind of skin lesion with 3 s integration time. Before the experiment, a standard tungsten lamp (RS-3, EG&G Gamma Scientific, USA) was used for calibrating the spectral response of the system, and the Raman spectrum of silicon (520 cm^−1^) was measured to calibrate the wavelength position.

### 3.3. Raman Data Processing

The WITec Project FOUR (WITec GmbH, Germany) was used to preprocess all the obtained datasets for band range selection, cosmic ray removal, 10 points Savitzky–Golay (SG) smoothing, and background subtraction. All the Raman spectra were normalized by their respective areas under the curves between 600 cm^−1^ and 1800 cm^−1^. In order to illustrate the best difference between selected bands, we employed one way ANOVA and Tukey’s honest significant difference (HSD) post hoc multiple test with a 5% significance level and had drawn a series of histograms. The difference between data is expressed by the probability caused by the sampling error, in which *p* < 0.05 means different and *p* < 0.01 indicates a significant difference.

## 4. Conclusions

Raman spectroscopy has a sound potential for providing a minimally invasive dermatological diagnosis of skin cancer. In this study, with H&E stained pathological images, the spectral variations in different lesion regions were described for a clear interpretation of biochemical variations during the SCC pathological progress by Raman microspectroscopy. The obtained results suggested that the peak intensity variation tendency of collagen, DNA, and lipids is concordant in both two different grades of SCC. The achieved results suggest the cancer field effect of skin carcinogenesis and provide vital reference data for clinical Raman diagnosis.

## Figures and Tables

**Table 1:** Tentative peak assignments for human skin tissue Raman spectra. Greek letters denote the type of vibrational mode (υ, stretching; δ, deformation).

Wavenumber (cm^−1^)	Tentative Peak Assignment	Refs.
720	υ (C-N) nucleotide peak or lipid/DNA	[18,19]
753	Symmetric breathing of tryptophan	[18,19]
853	υ (C-C) collagen proline ring	[10,20,21]
936	υ (C-C) collagen backbone/proline ring	[10,20,21]
1002	υ (C-C) aromatic symmetric ring breathing of phenylalanine	[22,23]
1032	C-N in-plane bending of phenylalanine	[24,25]
1040	υ (S-O) cysteic acid	[26,27]
1248	υ (CN) and δ (NH) amide III of collagen: proline rich	[11,28]
1305	δ (CH_2_) lipids/ceramide,	[22,29]
1327	CH_3_/CH_2_ wagging of nucleic acids	[19,30]
1450	δ (CH_2_) scissoring of proteins and lipids	[20,21,30]
1654	υ (C=C) Amide I (protein/lipid)	[28,29]
1661	υ (C=O) Amide I (collagen)	[21]

## Supplementary Materials

Supplementary File 1
