# Supplementary material for: Investigation on the Cancer Invasion and Metastasis of Skin Squamous Cell Carcinoma by Raman Spectroscopy
Source: Molecules. 2019 May 30;24(11):2059. doi: 10.3390/molecules24112059 (PMC6600666; doi:10.3390/molecules24112059)
Supplement: Supplementary File 1 [file molecules-24-02059-s001.pdf]

-Supporting Information-

Investigation on the cancer invasion and metastasis of skin squamous cell carcinoma by Raman spectroscopy

Xu Zhang [1,2], Fan Yu [1,2], Jie Li [1,2], Dongliang Song [1,2], Heping Li [1,2], Kaige Wang [1], Qingli He [2] and Shuang Wang [1,*]

[1] Institute of Photonics and Photon-Technology, Northwest University, Xi'an, Shaanxi 710069, China
[2] Department of Physics, Northwest University, Xi'an, Shaanxi 710069, China
* Correspondence: Shuang Wang, wsnwuphy@163.com, swang@nwu.edu.cn;

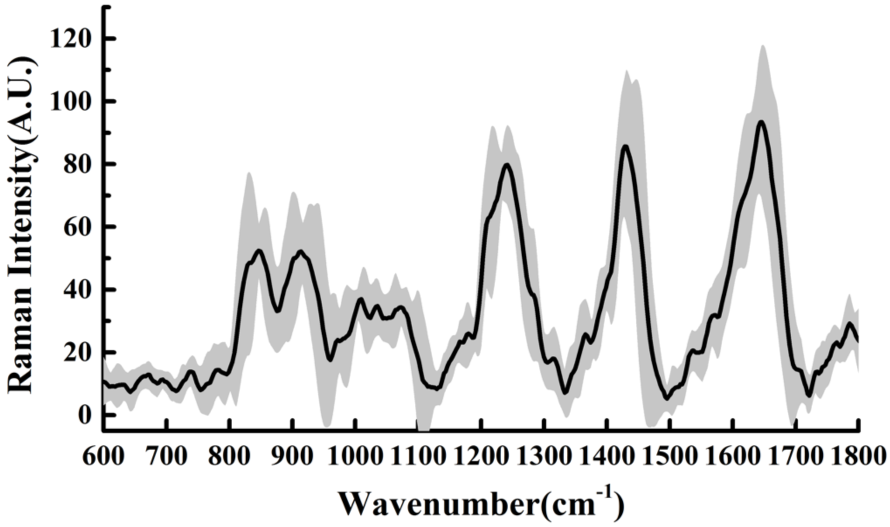

Mean Raman spectrum of tissue components from the neoplasia occurring in the dermis area, labeled as region IV in Figure 1(A). The standard deviation of all 30 randomly acquired spectra is marked in gray, which could prove and enhance our statistical findings and credibility.
